# Anodes for Li-ion batteries prepared from microcrystalline silicon and enabled by binder’s chemistry and pseudo-self-healing

**DOI:** 10.1038/s41598-020-70001-5

**Published:** 2020-08-06

**Authors:** Carl Erik Lie Foss, Stephan Müssig, Ann Mari Svensson, Preben J. S. Vie, Asbjørn Ulvestad, Jan Petter Mæhlen, Alexey Y. Koposov

**Affiliations:** 1 Institute for Energy Technology, P.O. Box 40, 2027 Kjeller, Norway; 2 Department of Material Science and Engineering, Norwegian University of Science and Technology (NTNU), Høgskoleringen 1, 7491 Trondheim, Norway

**Keywords:** Batteries, Batteries

## Abstract

Silicon, while suffering from major degradation issues, has been recognized as a next promising material to replace currently used graphite in the anodes of Li-ion batteries. Several pathways to mitigate the capacity fading of silicon has been proposed, including optimization of the electrode composition. Within the present work we evaluated different binder formulations to improve the long-term performance of the Li-ion batteries’ anodes based on industrial grade silicon (Si) which is typically characterized by a particle sizes ranging from 100 nm to 5.5 microns. The decrease of pH in a binder formulation was found to detrimental for the cycling performance of Si due to enhanced formation of an ester-type bonding between the carboxylic group of the binder and hydroxyl group on the Si surface as well as cross-linking. Furthermore, the present work was focused on the use of the industrial grade Si with very high loading of Si material (up to 80% by weight) to better highlight the effects of the surface chemistry of Si and its influence on the performance of Si-based anodes in Li-ion batteries. The tested system allowed to establish a *pseudo* self-healing effect that manifests itself through the restoration of the anode capacity by approximately 25% and initiates after approximately 20 cycles. The stabilization of the capacity is attributed to self-limiting lithiation process. Such effect is closely related to SEI formation and transport properties of an electrode prepared from silicon of industrial grade.

## Introduction

Silicon (Si) has been recognized as one of the most promising anode materials for Li-ion batteries due to its high gravimetric theoretical lithium storage capacity (3,579 mAh g^−1^)^1^, compared to conventionally used graphite anodes (372 mAh g^−1^)^2^, while also having a relatively low discharge voltage (the average delithiation voltage of Si is 0.4 V)^3^. However, the ability of Si to accommodate large amount of Li causes the material to undergo enormous volumetric changes during lithiation and delithiation^1,4–6^. Such morphology transformations occurring through the full lifecycle of the anode results in cracking of Si particles, formation of an immense surface stress, and, as a result, adhesion problems^7,8^. Particle detachment that follows cracking and fracturing leads to the loss of electrical contact, effectively decreasing the available active material and, therefore, diminished capacity. In addition, continuous cracking of Si particles occurring during cycling exposes fresh Si surface for further reaction with electrolyte. Thus, cracking of particles results in a continuous formation/repair of the passive layer known as the solid electrolyte interphase (SEI)^9,10^. As a consequence, the coulombic efficiency of the silicon-based electrodes is typically lower than for graphite electrodes.

Therefore, it is well recognized that mitigation of the particle fracturing can substantially extend the lifetime of a battery. Such approaches include nanostructuring^11–14^ and coating of the particles to minimize the damages caused by volumetric changes^15^. In addition to engineering of particles, the other components of an electrode, such as binders, could be efficiently optimized to accommodate expansion and contraction processes of Si while maintaining the integrity of the electrode^16^. The main roles of a binder in Si-based anodes are to control the particle’s expansion and shape changes as well as to keep the active particles together upon lithiation, thus, preserving the electrode integrity and effectively increasing cycle life. Several examples of binder systems have been shown to efficiently enhance the lifetime of Si-based anodes while also being inexpensive and readily available. Those include polyacrylic acid (PAA) and carboxymethyl cellulose (CMC)—two of the most common chemical systems that can be processed using water-based slurry chemistry^17^.

Earlier studies have demonstrated the importance of the processing conditions for extension of the lifetime and cyclability of Si-based anodes. For instance, the influence of the pH on the binding properties and cycle life for CMC is well documented^8,18–20^. This is mainly attributed to the acid-catalyzed formation of ester-type bonding by coupling of the carboxylic group of CMC and hydroxyl groups on the Si surface^20^. However, some studies indicate that absence of the covalent bonding between the CMC binder and silicon particles may be beneficial for extension of the lifetime of anode due to hydrogen bonding formation within the electrode^21^. PAA—another commonly used binder, has been thoroughly studied as binder in water and alcohol systems^22,23^ and in combination with Na-CMC^24,25^. The performance of PAA was also found to be dependent on the pH. It has been shown that treatment of PAA with LiOH/NaOH (at pH around 8) allows to reduce irreversible capacity^26^. PAA has been found to have better capacity retention compared to CMC when LiFSI salt was used for electrolyte formulation^27^. The performance of binder system also strongly depends on the selection of the silicon material and selected electrolyte. While the vast majority of the studies of binder chemistries were performed on nanostructured Si specifically designed to be used in LIBs, industrially relevant micron-size Si has received considerably less attention. Such Si could be easily produced in large amounts and could be easily implemented in the next generation of LIBs if the performance of such material could be improved. Therefore, within the present work the effect of the binder chemistries on the long-term behaviour of the anodes based on industrial grade Si particles (*Elkem, Silgrain e-Si 400*) is evaluated. Furthermore, the effect of the electrolyte additive’s, such as fluoroethylene carbonate (FEC), concentration on the electrode performance is studied.

## Experimental

### General

All manipulations with the Si particles were performed under ambient pressure and temperature. Unless specified, all chemicals used for the battery fabrications were purchased from Sigma-Aldrich and used without further purifications. The slurries were prepared with either a buffer solution (pH 3) prepared from citric acid and potassium hydroxide using deionized water (18 MΩ), or deionized water (18 MΩ), as a solvent. All cell assembly was carried out in Ar-filled glovebox (< 0.1 PPM O_2_ and < 0.1 PPM H_2_O). For SEM characterization, Si samples were dispersed in ethanol, ultrasonicated, and pipetted onto lacey carbon TEM grids. SEM analysis was performed on the TEM grids using a Hitachi S-4800 instrument operating at 30 kV equipped with a retractable transmission detector (low voltage STEM).

### Anode fabrication

The anodes were prepared by screen printing of slurries prepared according to the following general procedure. 100 mg of a binder material was added to either water or buffer solution and the resulting mixture was sonicated for at least 30 min to assist the binder dissolution. Then, 1,000 mg of Si particles (Elkem, Silgrain e-Si 400) and 150 mg of carbon back (Super C 65, Timcal) were added to the binder solution. The formulation was mixed with an IKA T-25 disperser for 30 min at 6,000 rpm. The ratio between the components was kept as 80:12:8 by weight and the ratio of solvent/powder was 2.5:1, unless specified otherwise. With a buffer this ratio becomes 73:11:7.5, as buffer accounts for about 8.5 wt% of a total mass. The slurry was then screen printed (using a 200/40/15 mesh) onto a 16 µm structured copper foil (SE-Cu58, Schlenk) and dried in air at room temperature for 24 h, followed by drying in a vacuum oven at 120 °C for 3 h. After drying, the electrodes were punched into 15 mm disks for further use in battery assembly. The silicon loadings of all tested electrodes were in the range of 0.905 ± 0.12 mg/cm^2^, to make the electrodes relevant in terms of commercial areal capacities (> 3 mAh/cm^2^).

### Battery fabrication and electrochemical characterization

The tested battery coin cells were assembled in a half-cell configuration using CR2032 stainless steel casings using Li foil counter electrodes (99.99%, LinYi Gelon LIB Co., 15 mm in diameter and 0.250 mm thick). Monolayer porous polypropylene film (Celgard 2400) was punched into 18 mm discs for use as separator. Both electrolytes studies in the present work were purchased from Solvionic—the electrolyte denoted herein as G1 consisted of 1 M LiPF_6_ in ethylene carbonate:propylene carbonate:dimethyl carbonate (EC:PC:DMC, 1:1:3 by volume) with 1 wt% VC and 5 wt% FEC as additives. The second electrolyte denoted herein as S1 consisted of 1.2 M LiPF_6_ in ethylene carbonate:ethyl methyl carbonate respectively (EC:EMC, 3:7 by volume), with 10 wt. % of fluoroethylene carbonate (FEC) and 2 wt. % of vinylene carbonate (VC) as additives. For each cell, 35 µL of electrolyte was used. The half-cells were tested using an Arbin BT-2000 cell tester and cycled galvanostatically between 0.05 and 1.0 V vs Li^+^/Li at the temperature of 25 °C, with two initial formation cycles at a rate of C/20 (179 mA/g_Si_) followed by continuous cycling at C/5 (716 mA/g_Si_), unless otherwise specified. Full cells were assembled with 1.0 mAh/cm^2^ Lithium-Iron-Phosphate (LFP) commercial cathodes from Customcells and cycled between 2.5 and 3.8 V vs. Li/Li^+^ at the temperature of 25 °C, with two initial formation cycles of C/20 (7.5 mA/g_Si_) followed by continuous cycling at C/5 (30 mA/g_Si_).

### Post-mortem analysis

For post-mortem analysis the half cells were assembled, cycled for 5 cycles with two initial formation cycles at a rate of C/20 (179 mA/g_Si_) followed by cycling at C/5 (716 mA/g_Si_). the cells were then disassembled, the anodes were washed with dimethyl carbonate (DMC) and imaged using SEM.

## Results and discussion

**Figure 1:**
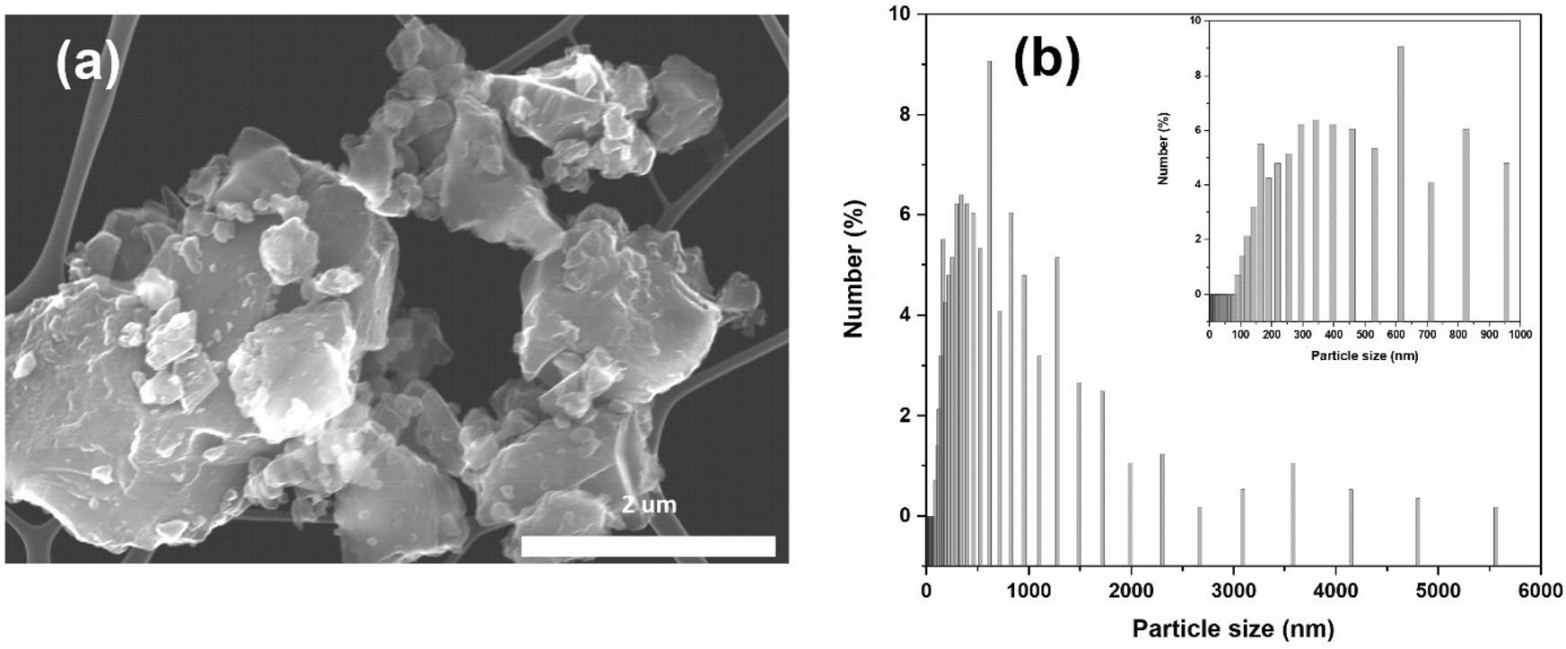
Low resolution SEM imaging of (**a**) Si microparticles used in the present work, (**b**) particle size distribution as estimated from SEM imaging, the inset shows detailed view of the area 0–1,000 nm.

A typical sample of the microcrystalline, industrial grade Si could be represented as an ensemble of polycrystalline particles with sizes ranging from 100 nm to 5.5 microns which function will depend on the binder and cycling conditions^28^. Scanning Electron Microscopy (SEM) imaging of a representative set of the Si particles utilized in this work is shown on the Fig. 1a next to the histogram illustrating the particles size distribution as shown on the Fig. 1b. Within the present work the electrodes were prepared with *very* high loading of Si particles (80% by weight) to better highlight the influence of the binder chemistry on the stability of the anode and the cell. The high Si loading and the large size of the Si particles resulted in non-uniform electrode morphology as can be seen on the Fig. S1 of Supporting Information. No noticeable differences in electrode morphology was observed when different binder formulations were used.

**Figure 2:**
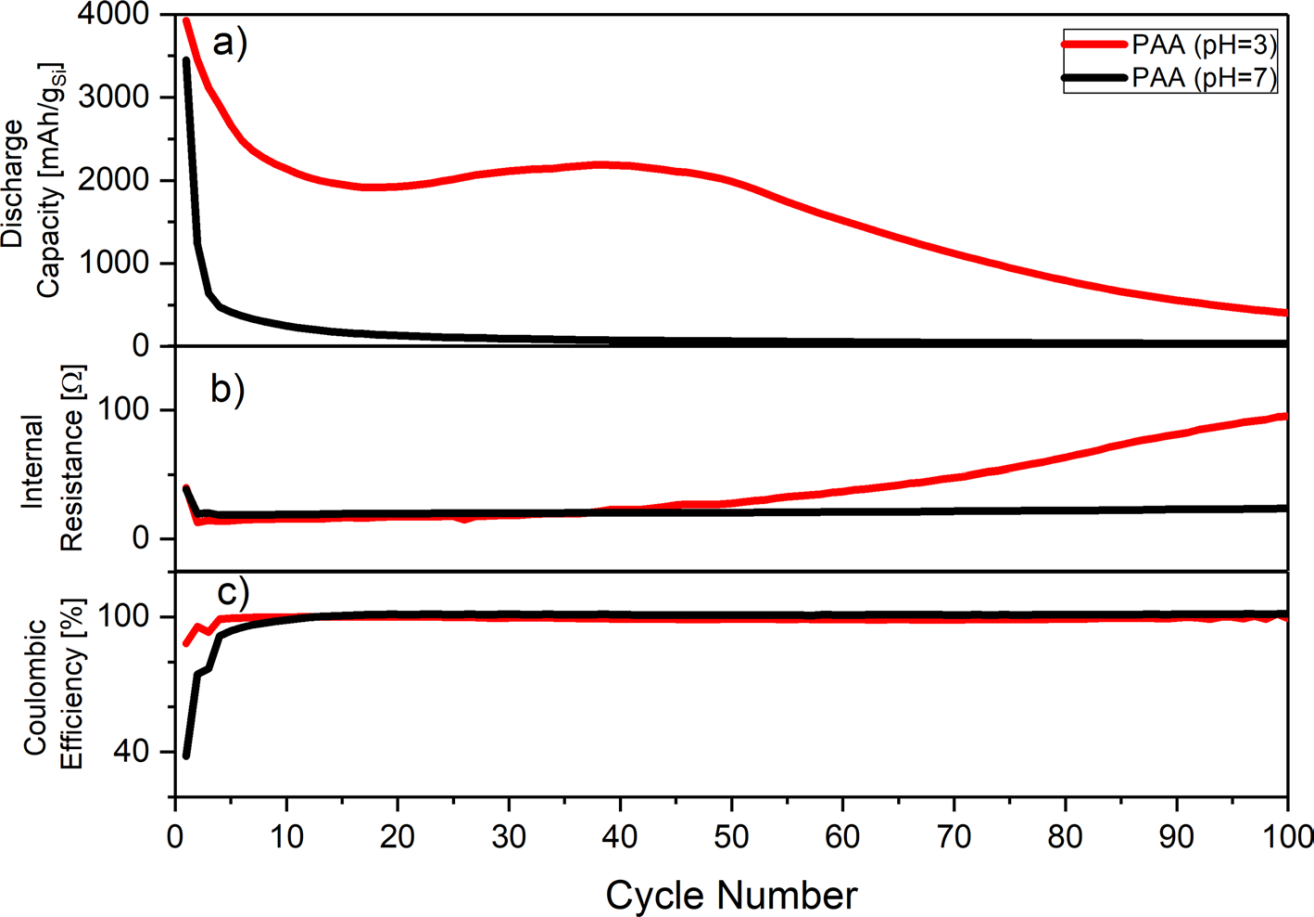
Cycling behavior of the anodes prepared at different pH evaluated using G1 electrolyte (5% FEC) in half-cell configuration: (**a**) discharge capacity; (**b**) internal resistance; (**c**) coulombic efficiency as a function of cycle number.

As obtained, the Si particles were processed to make electrodes and their electrochemical performance was evaluated in a half-cell configuration. The binders such as PAA, CMC and a combination of PAA/CMC (1:1 weight ratio) processed at neutral pH (7) and low pH (pH of 3) These formulation chemistries (different binders at different pH) were evaluated in half-cell configurations using Li foil as counter electrodes using G1 electrolyte. For cycling of the fabricated cells galvanostatic conditions at room temperature were applied, and the corresponding electrochemical performances (discharge capacity vs. cycle number) are shown on Fig. 2 which summarizes the behavior of the anodes prepared using PAA as a binder.

The cycling behavior of the electrodes unambiguously demonstrated the importance of the pH during processing of the anodes fabricated from microcrystalline Si and PAA. While the benefits of the low processing pH for some types of Si are well documented^27^, for the microcrystalline Si used in the present work the effect is dramatic. Specifically, slurry processing at neutral pH leads to largely non-functioning anode where complete capacity fading occurs after completion of formation cycles. The formation cycles for such anodes are also characterized by a low Coulombic efficiency (below 40% for the first cycle, Table S1 of Supporting Information). While the capacity/voltage through initial charging are almost the same for anodes processed at neutral and low pH, the anodes prepared at neutral pH demonstrated large irreversible capacity which is most likely due to the detachment of the active material from the electrode (Fig. S2 of Supporting Information). On the contrary, the anodes prepared at low pH have demonstrated a behavior rather common for that type of Si material. Thus, similarly to CMC binders^28^, the slurry preparation at pH 3 has a tremendous impact for the PAA-Si system, particularly when large Si particles are used.

It is well recognized in the literature that the use of different binder formulations will promote different binding modes^17^. Specifically, the processing condition such as pH of the slurries needed for the electrode casting will have a great influence on bonds formation between the carboxylic group of a binder and hydroxyls groups on the surface of Si particle if a binder with carboxylate moieties is selected. Lower pH promotes the formation of ester-type bonding with formation of Si–O–C fragments, therefore connecting Si particle with the rest of the electrode and maintaining its integrity. However, even carboxylate-based binders are different—PAA is a linear polymer while CMC is more prone to cross-linking^29^, thus the mechanical robustness and flexibility of electrodes made from different binders will be different. This factor is particularly important during expansion and contraction of Si associated with the lithiation/delithiation processes. However, within the present work no significant difference has been observed between CMC and PAA binder systems and processing pH had a significantly more noticeable effect on the electrode performance (Fig. S3 of Supporting Information). The high loading of Si might be the primary factor behind this observation.

**Figure 3:**
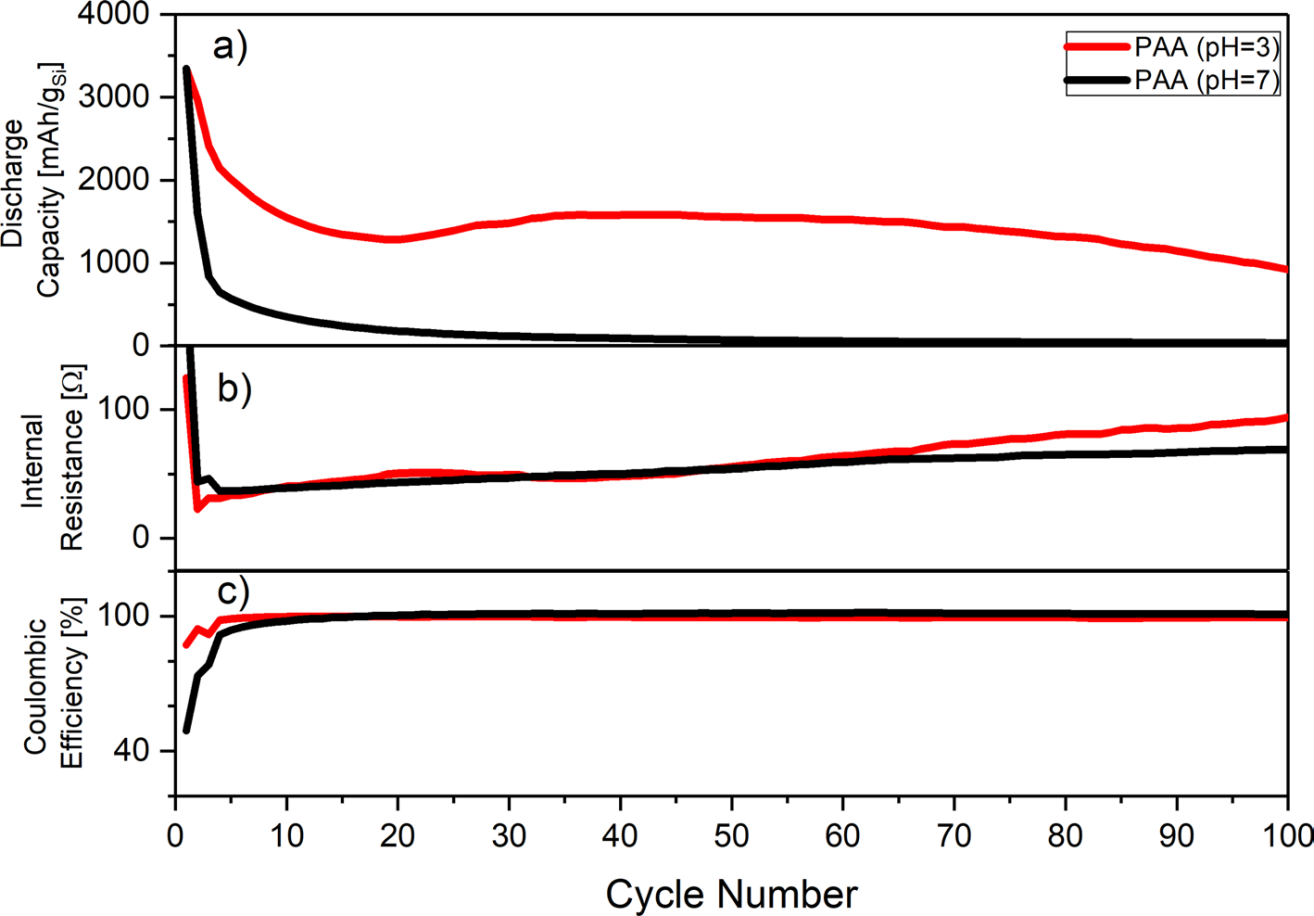
Cycling behavior of the anodes prepared using at pH evaluated using S1 electrolyte (10 wt% FEC) in half-cell configuration. (**a**) discharge capacity; (**b**) internal resistance; (**c**) coulombic efficiency as a function of cycle number.

The presence of FEC is known to partially mitigate the degradation issues for Si-based anodes^30–32^. The concentration of FEC essentially allows to manage the lifetime of the anode, by participating and influencing SEI formation, making it more flexible and, therefore, allowing it to accommodate the shape changing of Si during cycling. Therefore, it is expected that an increased amount of FEC will also improve the stability of the Si-based anodes fabricated from microcrystalline Si. This would be of particular importance to test on the electrodes prepared under neutral pH to understand if FEC can alter the effect of the binder’s chemistry. Thus, the electrolyte S1 containing 10 wt% of FEC was tested in the half-cell configuration with PAA (the electrochemical performance is shown on Fig. 3) and in combination with different binder chemistries as shown in the supplementary information (Fig. S4).

Similarly to the results observed for FEC-poor electrolyte (G1), the pH processing of the electrodes have a tremendous effect on the lifetime and overall performance of the electrodes: the electrodes processed at neutral pH were not functional beyond formation cycles despite the presence of extra amount of FEC, unlike in abovementioned examples reported in the literature. Specifically, the pH of processing controls not only the lifetime of an electrode but its ability to function beyond the formation cycles. We believe such difference in behavior is due to structural stability of the electrodes. The high loading of Si and size of the particles substantially differentiate the studied system from the majority of abovementioned Si systems evaluated previously. The secondary bonding between the binder and hydroxyl groups on the Si surface obtained at neutral pH is no longer sufficient to maintain the structural integrity of the electrodes leading to the detachment of the Si particles from the electrodes. Such detachment leads to rapid capacity fading and electrode failure. The stronger covalent binding delivered by formation of the ester bonding between the carboxylic groups of binders and hydroxyls at the surface obtained at low pH allows to keep the electrode structure allowing the electrodes to behave accordingly. To verify that a set of the cells was fabricated and cycled for 5 cycles, after which the cells were disassembled and examined by SEM. The corresponding images are shown on the Fig. S3 of Supporting Information. Briefly, while the electrodes prepared using low pH represent a usual view of the mildly cycled electrodes with all the components fully interconnected and covered by SEI, the electrodes prepared at neutral pH could viewed as a mechanical mixture of the components without any clear interconnections. Full cells were assembled to demonstrate the effect of pH without the potential influence from the lithium metal counter electrode. These cells (the cycling performance is shown on Fig. S4 of Supporting Information) show a clear degradation difference between the two systems, as in half cells. However, the degradation seems to be less severe in full cells compared to half cells. The reason for such difference is because in full cells the anode capacity is over dimensioned compared to the cathode to prevent lithium plating, and, therefore, the resulting stress on the anode is far less compared to half cells systems.

**Figure 4:**
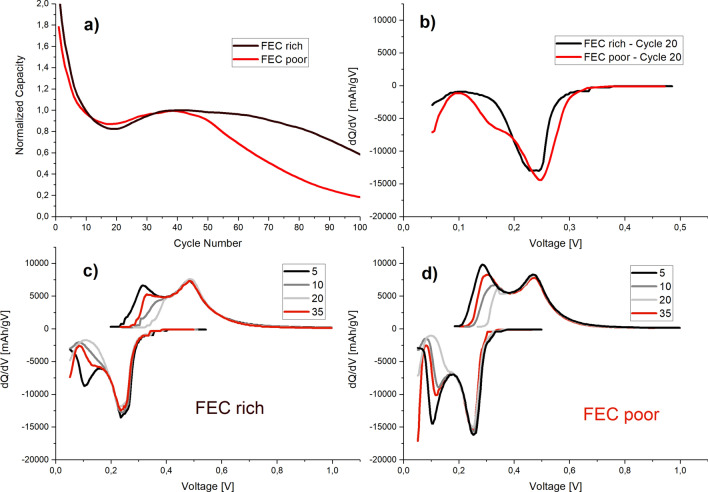
Illustration of the *pseudo* self-healing effect in the microcrystalline Si anodes prepared with PAA binder at low pH formulations measured with different electrolytes FEC-poor (G1) and FEC-rich (S1): (**a**) normalized capacity with respect to peak recovery capacity; (**b**) differential capacity at cycle 20; (**c**) differential capacity plots at 5, 10, 20 and 35th cycles using S1 (FEC rich) electrolyte; (**d**) differential capacity plots at 5, 10, 20 and 35th cycles using G1 (FEC poor) electrolyte.

The cycling behavior for the anodes prepared at low pH demonstrated an unusual behavior—the restoration of the discharge capacity of the functioning electrodes which could be clearly seen in Figs. 2a and 3a. This electrode’ *pseudo* self-healing phenomena represents itself through the restoration of the anode capacity by approximately 25% and starts after approximately 20 cycles. The effect of the *pseudo* self-healing clearly depends on the electrolyte and is more pronounced with higher concentrations of FEC, which suggests that the origin of this effect is SEI-related (Fig. 4a). More extreme situation is observed when CMC is used as a binder—the *pseudo* self-healing effect is barely noticeable for low FEC concentration, while for high concentrations of FEC it is comparable with PAA or a combination of CMC/PAA (Figs. S5 and S6 of supporting information). The SEI formation depends on the type of binder, and was shown to be different for the PAA binder compared to the CMC binder as it was shown in^27^. After reaching the maximum at approximately 50 cycles the capacity of the anode starts to decay in a fashion common for Si-based anodes as highlighted on the Fig. 4a.

The classical behavior of Si at the early stages is represented by two peaks in the differential capacity plot, corresponding to the subsequent formation of two amorphous phases during lithiation process (*a*-Li_2_Si approximately around 0.25 V and *a*-Li_3.75_Si at lower voltages)^33^ . Similar behavior is observed during delithiation process as well. The evaluation of the differential capacity allows to interpret and understand the observed *pseudo* self-healing effect, particularly for the FEC rich electrolyte. The capacity fading (shown on Fig. 4a) is correlated with the disappearance of the peak associated with the formation of the highly lithiated phase, self-limiting the lithiation of Si microparticles to a formation of Li_2_Si only (Fig. 4b. Both peaks are present at the early stages of cycling; the differential capacity curves for cycle 5 show two peaks for both the lithiation and the delithiation processes for both types of electrolytes (Fig. 4c,d, black lines) while for the subsequent cycles, the peak around 0.1 V corresponding to the formation of the highest lithiated phase gradually disappears. Then, after 20–30 cycles the peak corresponding to the formation of *a*-Li_3.75_Si reappears and helps to restore the capacity of the electrode, manifesting itself as *pseudo* self-healing mechanism. The similar situation is observed for the corresponding delithiation curves. The term *pseudo* is used to reflect the fact that no structural healing occurs in the silicon material, but rather an electrode structure undergoes changes that partially recover previously lost capacity.

The extent of the peak suppression strongly depends on the electrolyte composition, such as FEC concentration: for FEC-poor electrolyte the suppression of the lithiation is less pronounced, while for FEC-rich electrolytes the *a*-Li_3.75_Si formation is completely suppressed around cycle 20 (Fig. 4b). Similar behavior was observed when alternative binders were used, such as CMC (Figs. S7 and S8). However, for CMC-based electrodes the difference between electrolyte systems is even more pronounced than for PAA-based electrodes. This dependency on the electrolyte composition and binder allows to suggest that self-limiting lithiation is dictated by the SEI formation together with elasticity of a binder. The SEI buildup occurring through the initial cycles leads to limitation of the lithiation process, which could be viewed as self-limitation by the Si-based electrode. Such self-limitation could be explained if the SEI is dense and elastic enough to suppress the expansion of the particles during lithiation i.e. the associated strain buildup limits the diffusion of two-phase boundary between *a-*Li_2_Si and *a*-Li_3.75_Si. The high loading of Si in the electrodes which limits the space available for the expansion could also contribute to this effect. It is known that the presence of FEC changes the formation of SEI by promoting the formation of polymer which provides more uniform, dense and conformal coverage of the particle^34,35^. In the present case we assume that the active particle might be fully covered as SEI behaves as a protective coating as it builds up at the early stages. The larger amounts of FEC considered together with relatively low surface area of the microcrystalline Si allows to observe this effect regardless of the binder system.

The restoration of the capacity (*pseudo* self-healing) occurs due to gradual reappearance of the fully lithiated phase. The principles behind the restorations are opposite to those which led to self-limitation of lithiation—through extended cycling the SEI starts to break down allowing further expansion of the particles. That process reduces the effect of the self-limiting lithiation and restores the full capacity of silicon, gradually reintroducing the normal degradation behavior of silicon as was described in the earlier works. However, such self-limitation extends the lifetime of the electrode due to diminished fracturing of the particles particularly at the early stages. By limiting the lithiation, the overall volume expansion and strain on the electrode are reduced.

## Conclusions

In summary, the behaviour of the microcrystalline silicon is demonstrated as function of the binder’s chemistry. Specifically, processing the electrode formulation at low pH is crucial not only for the extension of the lifetime of Si, but for the electrode to have any practical functionality at all. Furthermore, the detailed analysis of the cycling behaviour of the electrodes prepared from the microcrystalline Si at high loading (80%) demonstrated temporary restoration of the capacity, denoted herein as *pseudo* self-healing effect. Such an effect is explained through self-limited lithiation occurring at the earlier stages of cycling. Such self-limiting lithiation was found to be dependent on the concentration of FEC in the electrolyte: at high FEC concentrations (10%) the lithiation is limited to the formation of Li_2_Si. *Within present work high concentration of FEC allowed us to highlight this effect.* High loading of the active material possibly contributes to this effect as well. The restoration of the capacity occurs due to SEI fracturing and opening the pathways for further lithiation.

## Supplementary information

Supplementary Information.

## Data Availability

The datasets generated during and/or analyzed during the current study are available from the corresponding author on reasonable request.
