# Supplementary material for: Anodes for Li-ion batteries prepared from microcrystalline silicon and enabled by binder’s chemistry and pseudo-self-healing
Source: Sci Rep. 2020 Aug 6;10:13193. doi: 10.1038/s41598-020-70001-5 (PMC7413325; doi:10.1038/s41598-020-70001-5)
Supplement: Supplementary file 1 — Supplementary Information. [file 41598_2020_70001_MOESM1_ESM.pdf]

SUPPLEMENTARY INFORMATION

Anodes for Li-ion batteries prepared from microcrystalline silicon and enabled by binder's chemistry and pseudo-self-healing.

Carl Erik Lie Foss [a,*], Stephan Müssig [a], Ann Mari Svensson [b], Preben J. Vie [a], Asbjørn Ulvestad [a], Jan Petter Mæhlen [a] and Alexey Y. Koposov [a,*]

a Institute for Energy Technology, P.O. Box 40, NO-2027 Kjeller, Norway

b Department of Material Science and Engineering, Norwegian University of Science and Technology (NTNU), Høgskoleringen 1, NO-7491, Trondheim, Norway

* Corresponding authors

TABLE S1 Summary of 1st cycle CE for different binders and electrolytes studies in the present work

Binder system	1st cycle CE	pH	Electrolyte	Average Loading (mg/cm^2)
**Silicon electrode**				
PAA	49.0	7	S1	0.877
PAA	38.1	7	G1	0.860
CMC + PAA	66.7	7	S1	0.909
PAA	87.3	3	S1	0.968
PAA	88.3	3	G1	0.698
CMC	88.7	3	S1	0.805
CMC	86.8	3	G1	0.762
CMC + PAA	87.5	3	S1	0.941

G1 electrolyte consists of or 1M $LiPF_6$ in 1:1:3 ethylene carbonate:propylene carbonate:dimethyl carbonate (EC:PC:DMC, by volume) with 1 wt.% VC and 5 wt.% FEC as additives.

S1 electrolyte consists of 1.2 M $LiPF_6$ in 3:7 ethylene carbonate:ethyl methyl carbonate respectively (EC:EMC, by volume), with 10 wt. % of fluoroethylene carbonate (FEC) and 2 wt. % of vinylene carbonate (VC) as additives.

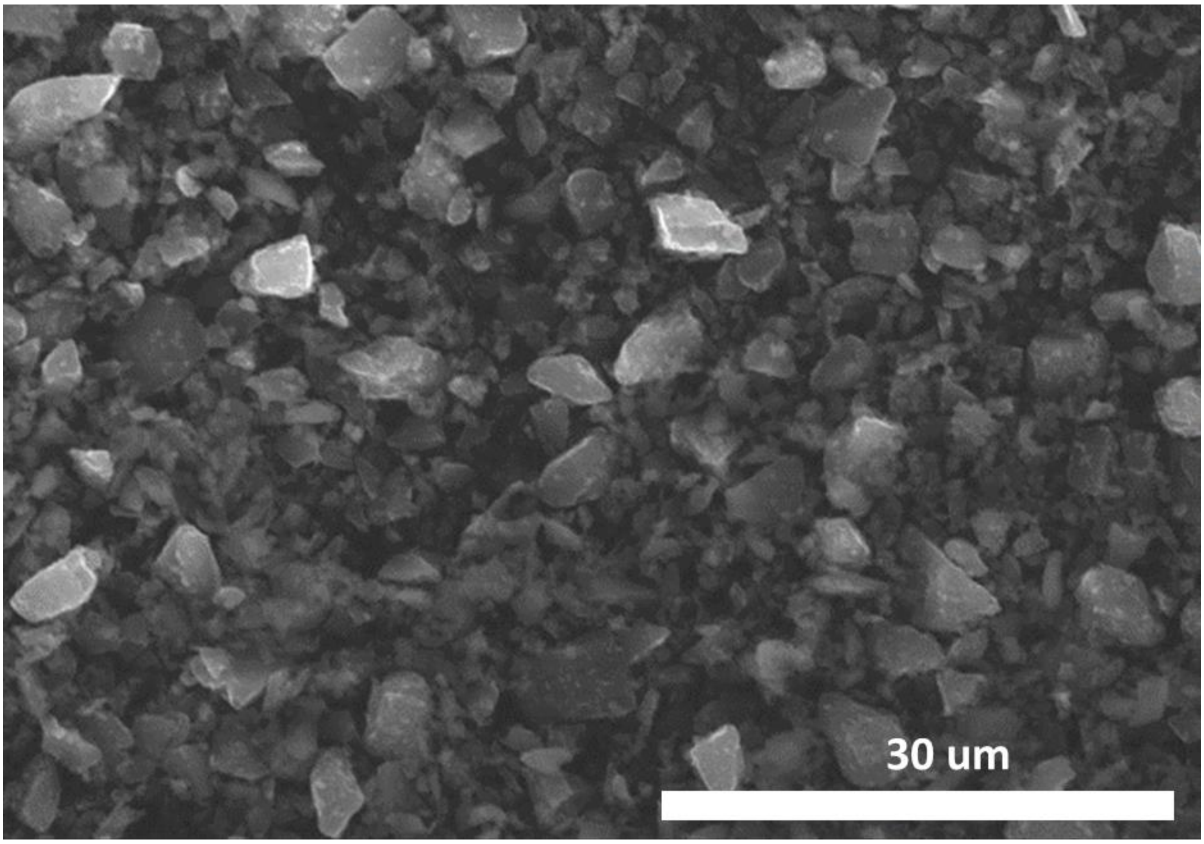

Figure S1. Low resolution SEM imaging of a top view of the anode fabricated from microparticles of Si.

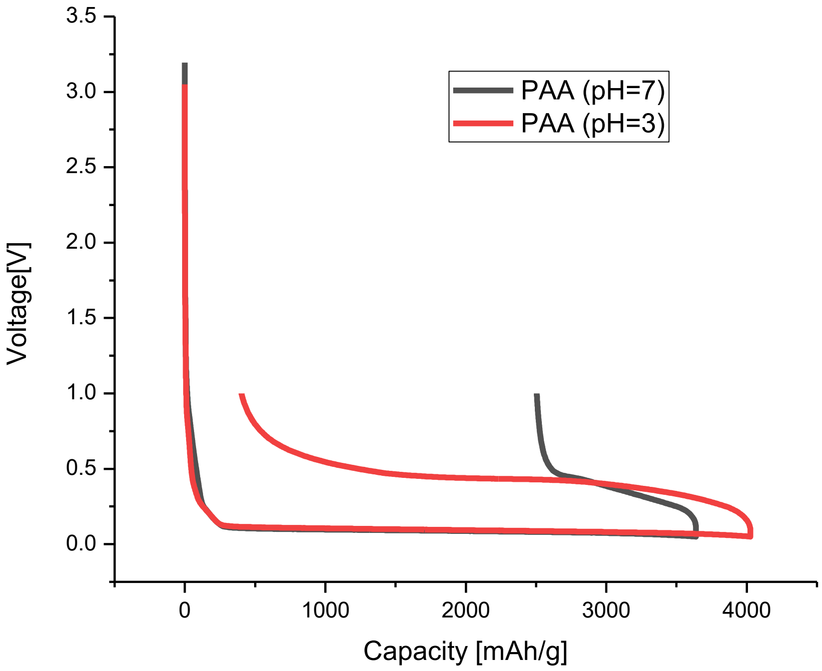

Figure S2. Voltage capacity curves for the anodes fabricated PAA processed at pH = 3 and pH = 7 (measured in half-cell configuration using Li foil as counter electrode and G1 electrolyte).

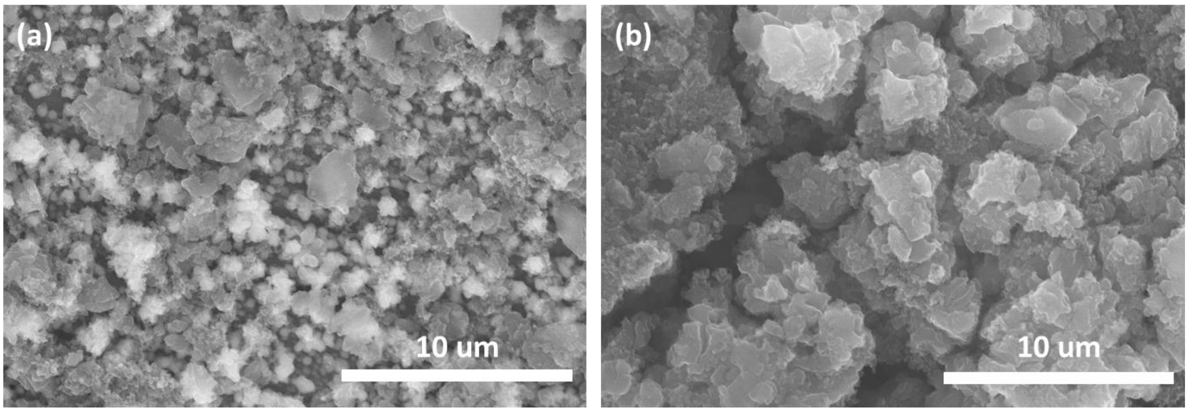

Figure S3. Post-mortem SEM imaging of the PAA-based electrodes after 5 cycles: a) fabricated at neutral pH, b) fabricated at pH=3.

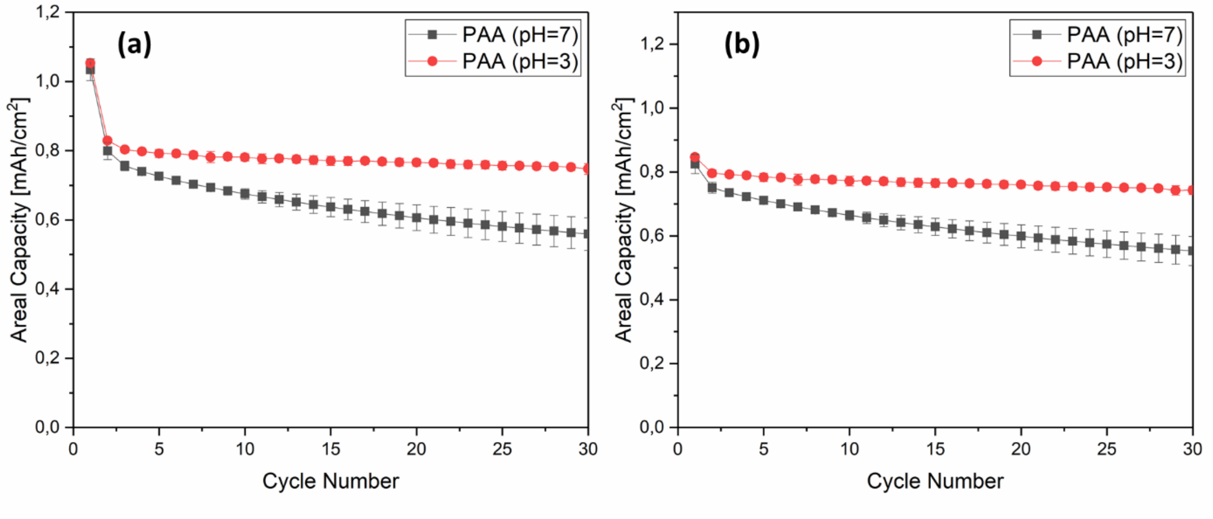

Figure S4. Lithiation capacity (a) and delithiation capacity (b) of anodes tested in the full cells.

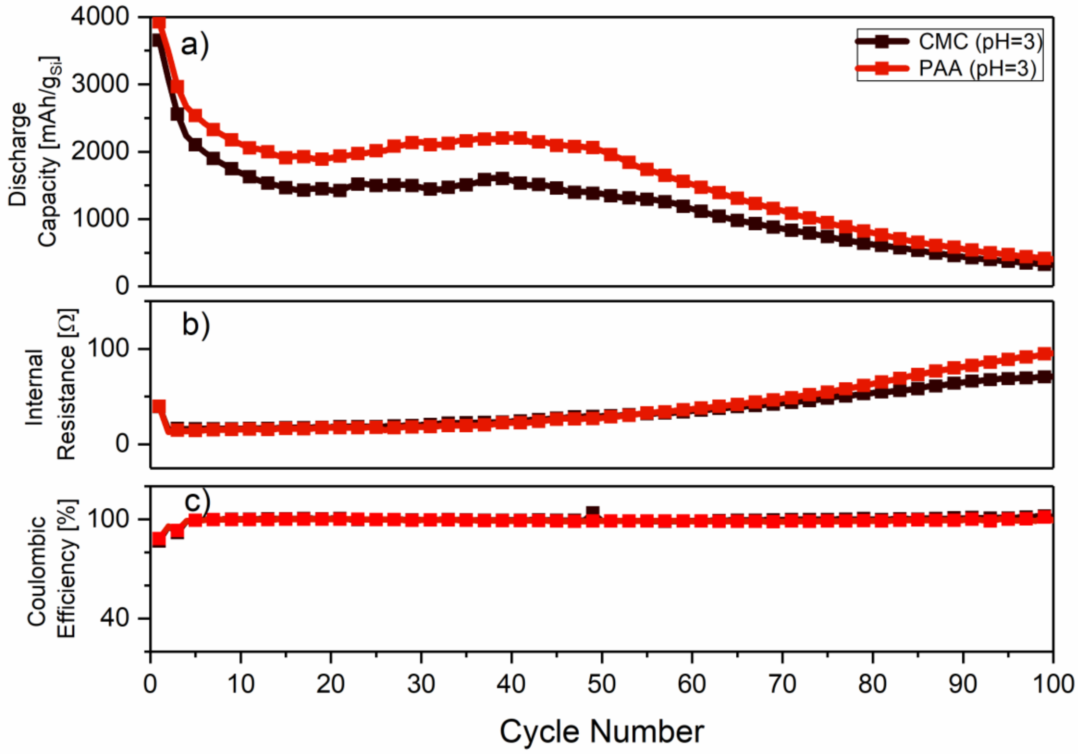

Figure S5. Comparison of the electrochemical performance for the anode fabricated using CMC and PAA processed at pH= 3 (measured in half-cell configuration using Li foil as counter electrode and G1 electrolyte).

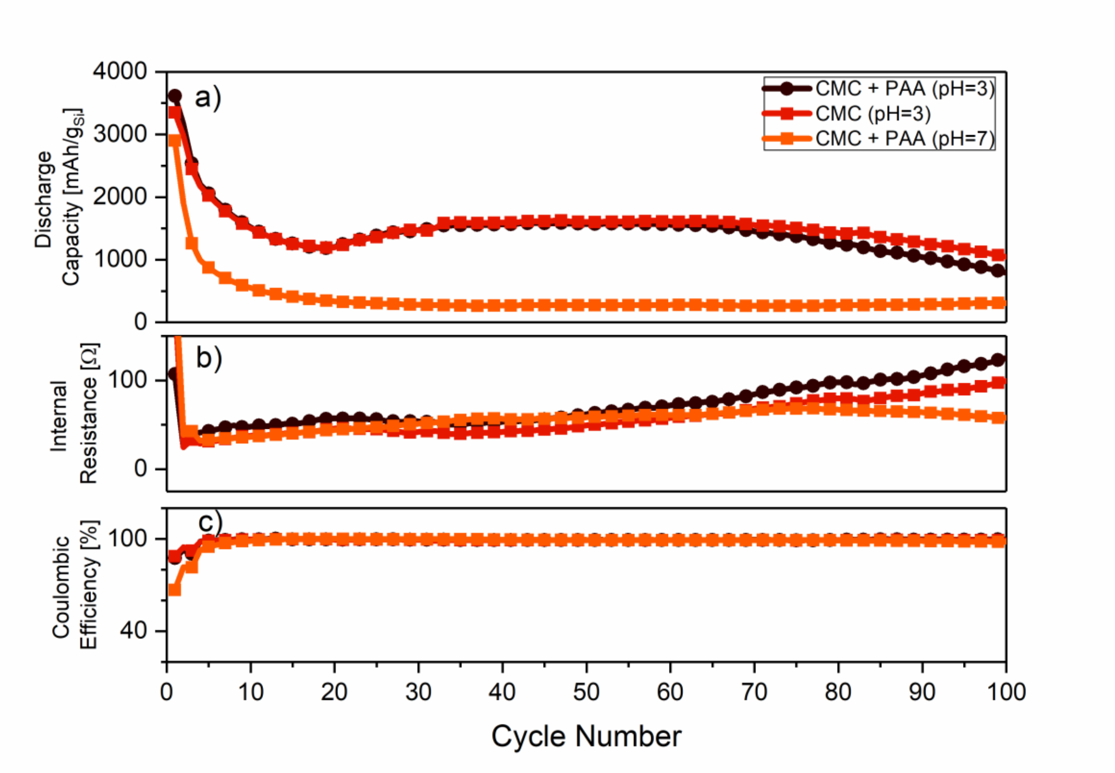

Figure S6. Comparison of the electrochemical performance for the anodes fabricated using CMC and a combination of CMC+PAA (50/50) processed at pH= 3 with anode fabricated using a combination of CMC+PAA (50/50) processed at pH= 7 (measured in half-cell configuration using Li foil as counter electrode and S1 electrolyte).

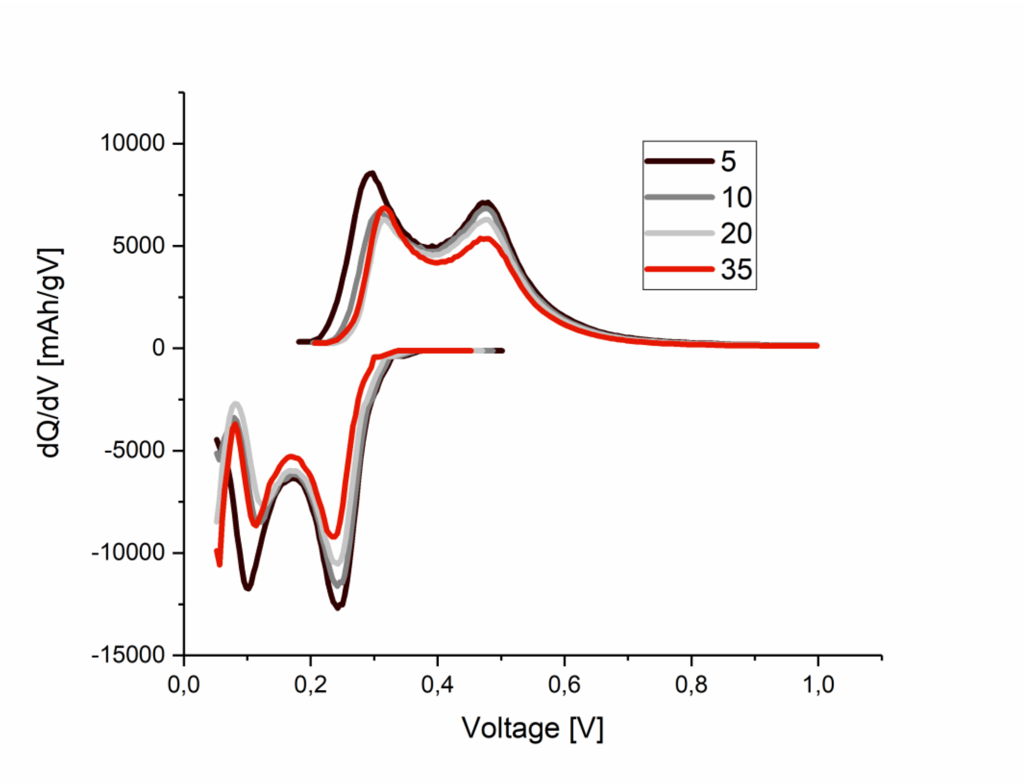

Figure S7. Differential capacity measurements for anode prepared using CMC processed at pH=3 with G1 electrolyte (FEC poor).

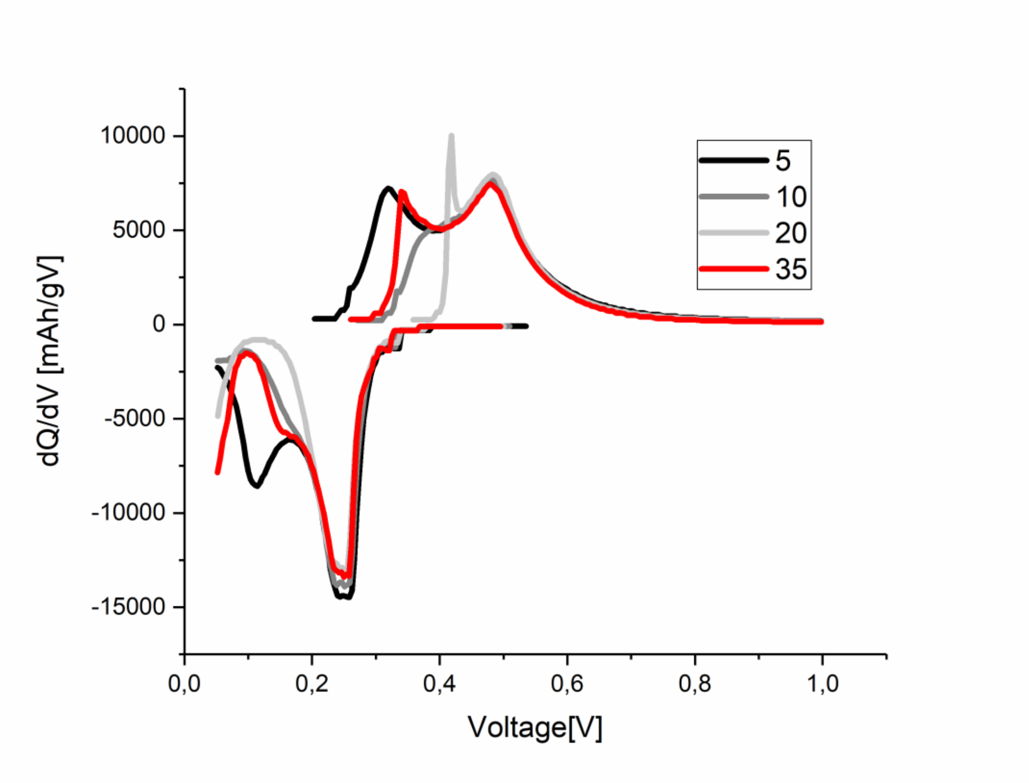

Figure S8. Differential capacity measurements for anode prepared using CMC processed at pH=3 with S1 electrolyte (FEC rich).
